# Global MicroRNA Expression Profiling of High-Risk ER+ Breast Cancers from Patients Receiving Adjuvant Tamoxifen Mono-Therapy: A DBCG Study

**DOI:** 10.1371/journal.pone.0036170

**Published:** 2012-05-18

**Authors:** Maria B. Lyng, Anne-Vibeke Lænkholm, Rolf Søkilde, Karina H. Gravgaard, Thomas Litman, Henrik J. Ditzel

**Affiliations:** 1 Institute of Molecular Medicine, University of Southern Denmark, Odense, Denmark; 2 Department of Pathology, Slagelse Hospital, Slagelse, Denmark; 3 Department of Biomarker Discovery, Exiqon A/S, Vedbæk, Denmark; 4 Department of Oncology, Odense University Hospital, Odense, Denmark; University of Pennsylvania School of Medicine, United States of America

## Abstract

**Purpose:**

Despite the benefits of estrogen receptor (ER)-targeted endocrine therapies in breast cancer, many tumors develop resistance. MicroRNAs (miRNAs) have been suggested as promising biomarkers and we here evaluated whether a miRNA profile could be identified, sub-grouping ER+ breast cancer patients treated with adjuvant Tamoxifen with regards to probability of recurrence.

**Experimental Design:**

Global miRNA analysis was performed on 152 ER+ primary tumors from high-risk breast cancer patients with an initial discovery set of 52 patients, followed by two independent test sets (N = 60 and N = 40). All patients had received adjuvant Tamoxifen as mono-therapy (median clinical follow-up: 4.6 years) and half had developed distant recurrence (median time-to-recurrence: 3.5 years). MiRNA expression was examined by unsupervised hierarchical clustering and supervised analysis, including clinical parameters as co-variables.

**Results:**

The discovery set identified 10 highly significant miRNAs that discriminated between the patient samples according to outcome. However, the subsequent two independent test sets did not confirm the predictive potential of these miRNAs. A significant correlation was identified between miR-7 and the tumor grade. Investigation of the microRNAs with the most variable expression between patients in different runs yielded a list of 31 microRNAs, eight of which are associated with stem cell characteristics.

**Conclusions:**

Based on the large sample size, our data strongly suggests that there is no single miRNA profile predictive of outcome following adjuvant Tamoxifen treatment in a broad cohort of ER+ breast cancer patients. We identified a sub-group of Tamoxifen-treated breast cancer patients with miRNA-expressing tumors associated with cancer stem cell characteristics.

## Introduction

Approximately 85% of breast carcinomas are estrogen receptor (alpha) positive (ER+), rendering these patients eligible for endocrine treatment with aromatase inhibitors (AIs) or Tamoxifen [1]. Adjuvant treatment with Tamoxifen significantly reduces the risk of recurrence and death in all age groups of ER+ patients. A meta-analysis of 21,457 women with breast cancer included in 20 trials of adjuvant Tamoxifen therapy showed a reduction of 15-year breast cancer mortality rates by at least a third [2].

Although Tamoxifen is of great benefit for many patients, recurrence occurs in approximately 30% after 15-years of follow-up [2]. Consequently, ongoing development of drugs for ER+ breast cancer has led to the development of third generation AIs, such as the non-steroidal agents Anastrazol and Letrozol and the steroidal agent Exemestane, which have increased efficacy compared to Tamoxifen in post-menopausal women [3]–[5]. Despite overall superiority of the AIs, Tamoxifen is still the recommended treatment modality for pre-menopausal breast cancer patients and patients resistant to AIs. In addition, the side-effect profile of the drugs differs, and some patients may not be candidates for treatment with a given drug due to co-morbidities. It is, therefore, rational to maintain Tamoxifen as an adjuvant treatment option, but the AIs have increased the need for more precise stratification of patients to ensure optimal patient care and the best use of health care budgets.

Micro-RNAs (miRNAs) are a class of non-coding, short RNAs (an average of 22 nucleotides) that function as post-transcriptional regulators by targeting mRNAs and causing either inhibition of translation or degradation of mRNA [6]. In essence, miRNAs add an extra level of regulation to gene expression, and studies are rapidly emerging on their role in diseases, including cancer. Their involvement in cancer is supported by an early finding that >50% of the miRNAs reside in cancer-associated chromosomal regions, e.g. regions of loss-of-heterozygocity, or common fragile sites [7].

Various studies have identified miRNAs that may be involved in ER regulation, and this mechanism has been proposed to be involved in the varying clinical benefits of Tamoxifen. MiR-206 was the first miRNA reported to be in a feedback loop with ERα [8]. To date, around 15 miRNAs have been identified that regulate the protein expression of ERα either directly or indirectly through interacting proteins, whereas the expression of three miRNAs (miR-206, miR-21 and miR-17∼92) has been found to be regulated by ERα/-β [9]. All of these studies were done using cell lines. The first report investigating Tamoxifen was on the hepato-carcinogenic effect of Tamoxifen in rats, finding an up-regulation of miR-17∼92, miR-206a and miR-34 in the liver after long-term exposure (24 weeks) to Tamoxifen [10].

Only a few studies have directly examined the role of miRNAs in Tamoxifen resistance, the vast majority of which were conducted using cell lines. These studies have yielded a list of miRNAs that may potentially be involved in resistance; miR-128a [11], miR-181, miR-489, miR-21 and miR-342 [12], miR-15a, miR-16 [13] and miR-101 [14].

Studies investigating patient material have been small and used clinical tumor material different that that used in the present study (primary tumors from patients who received Tamoxifen mono-therapy in the adjuvant setting). In one study, the miR-221/-222 cluster was found in cell lines to negatively regulate the ERα and was subsequently identified in 4/16 ER+ patients vs. 13/25 ER- patients. In addition, miR-221/-222 has been found to have higher expression in HER2-positive tumor samples, which are associated with poor outcome in Tamoxifen-treated patients (the ER status was not provided) [12].

In another study, miR-30c was identified as an independent predictor of Tamoxifen efficacy in advanced breast cancer patients. This patient population had already developed metastasis prior to the onset of treatment and the benefit of treatment was measured as an objective response according to the REMARK criteria [15]. Another recent study showed that miR-210 was associated with outcome in 56 untreated breast cancer patients, thereby providing a marker of breast cancer aggressiveness. This miRNA was confirmed to predict outcome in a treated population of 89 Tamoxifen-treated ER+ breast cancer patients [16]. Finally, a small study examining 15 post-menopausal, ER+ breast cancer patients receiving Exemestane (an AI) and Tamoxifen for 4 months prior to surgery found up-regulation of a panel of miRNAs after treatment (miR-21, miR181b, miR-26a/26b, miR-27b and miR-23b), providing insight into the effect of Exemestane and Tamoxifen on miRNA expression [17]. However, this study did not report a possible association between pre-treatment miRNAs and benefit of treatment, likely due to the small sample size.

The intent of our study was to obtain biological information on miRNA expression in a large cohort of ER+ breast cancer patients enrolled in the endocrine DBCG-89c/-99c trials, in which patients had been treated with adjuvant Tamoxifen, and to examine whether miRNAs could sub-stratify this patient population with regard to outcome. LNA-enhanced microarrays were used to measure global miRNA expression in primary ER+ tumors from high-risk, post-menopausal, breast cancer patients.

## Methods

### Patient Material

All included patients underwent primary breast cancer surgery at Odense University Hospital and were enrolled in the endocrine protocols of the Danish Breast Cancer Co-operative Group (DBCG) 89c or 99c [18]. All patients had archival formalin-fixed, paraffin-embedded, (FFPE) tumor tissue stored in the dark at room temperature. Clinical information was obtained from the DBCG. Inclusion criteria: ER+ tumor, post-menopausal, mono-systemically treated with Tamoxifen for >3 months and diagnosed at least 5 years prior to initiation of the study. Furthermore, the tumor was only included if the content of tumor cells was >50, as assessed by haematoxylin and eosin (HE)-stained sections. Exclusion criteria: bilateral breast cancer, recurrence <3 months of diagnosis, treatment with adjuvant chemotherapy/AIs, secondary cancers (except for cancer cutis) and/or unavailable medical records. Follow-up, or disease-free survival (DFS), was defined as time between diagnosis and date of last flow sheet for patients without recurrence (denoted N), or date of recurrence (denoted R). Overall survival (OS) was defined as period from date of surgery to nationally registered death from any cause. The study was initiated with a discovery set consisting of 26/26 samples from patients experiencing recurrence/no recurrence, respectively, followed by two independent test sets of 30/30 and 19/21 samples, respectively, from patients experiencing recurrence/no recurrence. The clinical characteristics are listed in Table 1. A power calculation was made to determine the number of samples needed to assure a statistically significant conclusion could be drawn. The power calculation was based on the following parameters: desired power 0.80, 2-fold expected difference, per-gene alpha 0.002, standard deviation estimate 0.7. The minimum number of patient samples per group/set was determined to be 16, and no further gain was likely at 30 or more patients per group/set. [20] Biological distribution between the recurrent vs. non-recurrent groups for each of the 3 sets was investigated by a 2-sided Students t-test. The study was approved by the Ethical Committee of Funen and Vejle County (VF20040064), The Danish Data Protection Agency (2004-53-0950) and the Danish Breast Cancer Cooperative Group (DBCG). The study was retrospective and we did not obtain informed consent from the participants involved in the study as approved by the Ethical Committee.

**Table 1:** Characteristics of included patients and their breast cancer tumor (N = 152).

	R_Disc._	N_Disc._	R_Test#1_	N_Test#2_	R_Test#2_	N_Test#2_
	(n = 26)	(n = 26)	(n = 30)	(n = 30)	(n = 19)	(n = 21)
**Age**	59.2	62.8	61.5	62.7	59.1	60
Avg. (range), years	(48–73)	(49–74)	(49–70)	(52–72)	(50–72)	(49–72)
**Size**	33.19	30.1	31.4	26.2	28.4	20.5
Avg. (range), mm	(12–85)	(14–95)	(7–80)	(8–58)	(8–65)	(10–45)
**Positive lymph node**	4.7	4.5	8.6	3.0	3.3	1.6
Avg (range)	(0–13)	(0–13)	(1–29)	(0–14)	(0–11)	(0–8)
**ER status** (avg. %/median %)^a^	91.4/95	85.9/100	77.9/90	83.9/83	82.4/92.5	83.1/90
Positive^b^	4	1	7	12	6	5
Negative	1	0	1	3	0	0
Unknown	0	2	1	3	1	0
**PgR status** (avg. %/median %)^a^	73.2/80	81.3/90	65/72.5	70.2/80	32.9/15	36.8/10
Positive^b^	3	1	9	9	1	0
Negative	6	7	11	5	2	6
Unknown	0	2	2	1	5	6
**Diagnosis** (IDC/ILC/unknown)	20/6/0	20/6/0	25/4/1	26/3/1	15/1/3	18/0/3
**Tamoxifen**	1.8	2.1	1.7	1.6	3.1	4.5
Avg (range), years	(0.3–5.0)	(0.7–5.0)	(0.8–5.2)	(0.8–5.3)	(1.7–5.0)	(2.7–5.0)
**TTR**	3.4	–	3.7	–	3.6	–
Avg (range), years	(0.7–8.7)		(0.8–14.9)		(0.8–8.7)	

a the average and median were calculated only for the tumors defined as positive, i.e. staining was observed in ≥ 10% of tumor cells by immunohistochemistry.

b If the actual percentage was not provided, patients were deemed positive if ER staining was observed in ≥ 10% of tumor cells by immunohistochemistry and/or target protein (ER or PgR) was >10 fmol/mg total protein as determined by biochemistry.

c the 5 ER- tumors had a PgR status of 90%, 50%, 90%, 80% and IHC+ (i.e. >10%) respectively.

Abbreviations: R: patients with recurrence. N: patients without recurrence. Disc.: Discovery set. Test#1: Test set#1. Test#2: Test set#2. Avg: average IDC: invasive ductal carcinoma. ILC: invasive lobular carcinoma. TTR: time to recurrence.

### RNA Isolation, Labeling, Hybridization, and Scanning

Five sections of 10 µm were de-paraffinized and total RNA was extracted with Roche high pure kit according to the manufacturer’s protocol (Roche, Basel, Switzerland). RNA quality and quantity was determined by NanoDrop spectrophotometer (Thermo Scientific, Wilmington, DE, USA). A common reference design wherein the common reference contained an equimolar mixture of total RNA from all samples per set was applied to allow both one and two-channel analysis of the data. The sample channel was labeled with Hy3™ dye, while the reference channel was labeled with Hy5™ dye according to the manufacturer’s recommendation (Exiqon A/S, Vedbaek, Denmark). Labeled RNA was hybridized overnight to miRCURY LNA™ microRNA arrays (see GSE37405 for details on the array features) on a Tecan HS Pro 4800 hybridization station. The arrays contained >3300 different Tm normalized capture probes, all in tetraplicates, against all human and viral microRNAs annotated in miRBase 15, as well as a number of proprietary microRNAs not yet included in miRBase, including the corresponding pre-miRNAs. After hybridization, the rinsed and dried arrays were scanned in a DNA microarray scanner (Agilent, model G2565BA, California, USA).

### Data Processing and Statistical Analysis

The data was treated as one channel data using the Hy3 signal intensities, and as two-channel data using the Hy3/Hy5 ratios. We present only the one-channel data since the ratio analysis gave comparable results. Initially, background subtraction was performed in R v.2.2.1 using the backgroundCorrectmethod in the LIMMA (Linear Models for Microarary Data) package, which is available as part of the Bioconductor project (http://www.bioconductor.org). The intensity values were then log2-scaled and the median probe signals were quantile normalized. Thus, the statistical analysis is based on the quantile normalized log2-scaled Hy3 signals.

The discovery set was used to examine the differences between the N and R samples, and between other clinical variables (according to Table 1), using a Student’s T-test. Data were visualized by dynamic PCA (principal component analysis) and in heat-maps with two-way unsupervised hierarchical clustering using Qlucore Omics Explorer v.2.2 (Qlucore AB, Lund, Sweden). For DFS and OS analysis, Kaplan-Meier plots and the Cox proportional-hazards regression model were applied to all three sets, using the R package “survival”, investigating the 10 miRNAs identified in the discovery set. A step-forward ANOVA model (used to limit type 1 errors when comparing more than one mean) was used for pair-wise comparison of microRNA biomarkers to clinical characteristics (e.g. grade). Subsequently, the two independent test sets were analyzed separately using supervised clustering for N vs. R samples to examine whether the identified miRNAs overlapped between the 3 sets.

An unsupervised analysis to identify potentially important miRNAs not associated with known clinical parameters was conducted. For each of the 3 data sets, the standard deviation was calculated and the 100 most variable miRNAs, per set, was compared with the other sets, identifying miRNAs that overlapped in all 3 sets. The biological association of the identified miRNAs were investigated using the miR2Disease database (www.mir2disease.org) [19]. This is shown in Table S1, which also includes experimentally verified targets.

## Results

### Patients

All patients had received adjuvant Tamoxifen as mono-therapy and enrolled in the DBCG cohorts 89c or 99c [18], [20]. Initially, a database retrieval of patients enrolled in the aforementioned endocrine protocols at Odense University Hospital was conducted by the DBCG. Eligible patients fulfilling the inclusion/exclusion criteria were grouped according to recurrence status. In total, there were 246 patients without recurrence and 80 with recurrence. The former patients were consistently the limiting factor. The median clinical follow-up was 4.6 years (range 0.7–14.9 years), and the most recently diagnosed patient was from 2001. Patient and tumor characteristics are provided in Table 1.

Matching was chosen for the discovery set (N = 52), equally divided according to recurrence status (Table 1), to accentuate underlying biological differences. The two patient groups (recurrence/no recurrence) were paired according to strong prognostic factors influencing the probability of recurrence, including number of positive lymph nodes and size of the tumor as well as duration of Tamoxifen treatment. Age was not a criterion for matching as all patients were post-menopausal. Matching was conducted to the precise number of tumor-infiltrated lymph nodes, which restricted the number of eligible patient-pairs.

Two independent test sets were used to examine the findings of the discovery set and included the remainder of the 89c/99c enrolled patients randomly selected from the previously-mentioned DBCG database retrieval. The only criterion was that half of the patients should have recurrence while the other half should not (Table 1), leading to a distribution resembling the complete nationwide 89c cohort as observed by the histological subtype. The 89c cohort is representative of this study in that it enrolled high-risk, ER+, post-menopausal breast cancer patients, and approximately 75% of the studied patients were part of 89c [20].

### Microarray Analysis

Global miRNA analysis of the 152 primary breast cancer samples was performed using LNA-enhanced microarrays, which contained probes enabling detection of both mature- and pre-miRNAs based on the miRbase 15.0 database. All data is available in the GEO dataset (accession number: GSE37405). Initially, the performance of the hybridization/microarrays was evaluated by visual inspection and manual flagging of poor quality spots in addition to Imagene quality analysis. Inspection of spike expression levels as well as correlation between arrays was checked for labeling control.

**Figure 1:**
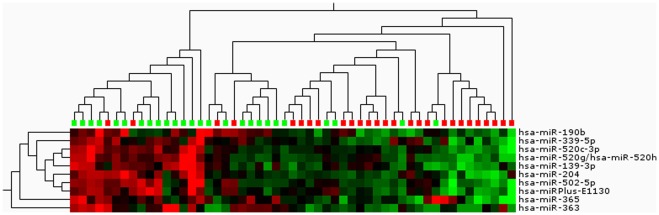
Heat-map of significantly differentially expressed miRNAs associated with outcome after adjuvant Tamoxifen treatment. Data is based on the discovery set (p<6.6e-4, FDR 2.5% and variance >0.1). The green symbols above the heat-map indicate samples from patients with no recurrence, whereas the red symbols indicate samples from patients with recurrence. The heat-map is a standardized intensity plot with the intensities ranging from −2 (green) to +2 (red).

The discovery set was used to examine the ability of miRNA to sub-group Tamoxifen-treated post-menopausal breast cancer patients according to outcome, which led to the identification of 10 highly significant miRNAs that distinguished recurrent from non-recurrent patients (p<6.6e-4, FDR 2.5%) (Fig. 1). The two test sets were also treated as individual sets to determine whether miRNAs could separate the N- and R-groups, and whether the identified miRNAs overlapped. Thirteen miRNAs (p<0.01, FDR 24%, variance >0.1) and 5 miRNAs (p<0.01, FDR 35% and variance >0.1) were identified for test sets #1 and #2, respectively, separating patient samples according to outcome (heat-maps are provided in Figure S1). None of the miRNAs identified in the test sets overlapped with those from the discovery set. The Discovery set consisted of matched patients according to previously detailed prognostic factors, whereas the 2 test sets were randomly grouped solely according to recurrence status. This lead to some biological differences wherein the number of tumor-infiltrated lymph nodes reached significance between the recurrent vs. non-recurrent groups, with p = 0.99, p<0.00001 and p = 0.05, for the discovery, Test set#1 and Test set #2, respectively.

**Figure 2:**
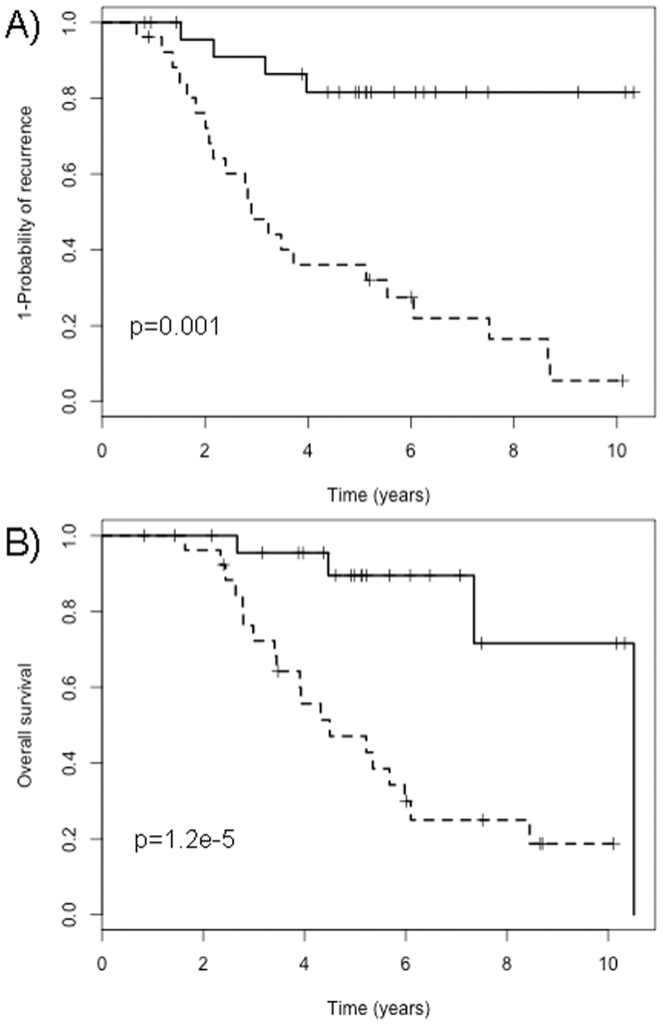
Kaplan-Meier plots of the 10 miRNAs identified in the discovery set. The bold line represents the patients with a good prognosis, whereas the dotted line represents the poor-prognosis patients. A) Probability of recurrence. B) Probability of overall survival.

The highly-significant miRNAs identified in the discovery set were analyzed by survival statistics, yielding a p-value of p_OS_ = 1.2e–05 and p_rec_ = 0.001 for overall survival (OS) and probability of recurrence (rec), respectively (Figure 2). Kaplan-Meier plots for the two test sets, applying the 10 identified miRNAs did not reach significance, with p_OS_ = 0.29 and p_rec_ = 0.89 for Test set #1, and p_OS_ = 0.3 and p_rec_ = 0.85 for Test set #2 (see Figure S2 and S3 for Kaplan-Meier plots).

**Figure 3:**
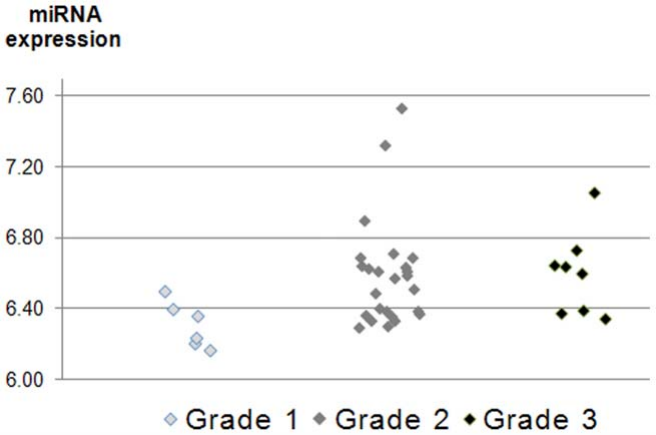
Association of miR-7 with tumor grade. Grade 1 vs. 3 and Grade 2 vs. 3: p = 0.01, and Grade 1 vs. 2: p = 0.02). N = 52 patients (Discovery set).

Furthermore, a supervised analysis according to standard clinical parameters (as listed in Table 1) was conducted along with several other clinical parameters such as grade, tumor content (percent tumor cells of the HE sections) and duration of treatment with Tamoxifen for the discovery set. A significant correlation was identified between miR-7 and the tumor grade (Figure 3), but otherwise no associations were found.

Unsupervised analysis was conducted to delineate miRNAs potentially related to biological functions. The standard deviation was used to identify the 100 most variable miRNAs per data set, unrelated to clinical characteristics. These miRNAs were compared across the 3 sets, leading to the identification of 31 miRNAs. The biological association of these 31 miRNAs is listed in Table S1 along with experimentally identified targets. Eight of these miRNAs, miR-141, miR-200a, miR-200b, miR-200c, let-7a, let-7b, let-7c and miR-205, have been reported to be associated with cancer stem cell characteristics, such as epithelial-mesenchymal transition (EMT) and self-renewal.

## Discussion

Endocrine resistance of ER+ breast cancer is a major clinical problem and the focus of intense research. The mechanism of endocrine resistance is not clearly understood and there is a strong need for predictive biomarkers that can guide clinicians to identify patients who will not benefit from adjuvant endocrine treatments, such as Tamoxifen and AI, and thus would need additional treatment. MiRNAs have been suggested as promising biomarkers, including potential markers of Tamoxifen resistance. Herein we report the first large-scale global miRNA expression study on primary tumors from high-risk, ER+, post-menopausal breast cancer patients who received adjuvant Tamoxifen mono-therapy. Our study showed that although a highly significant set of 10 miRNAs distinguishing patient samples according to outcome were identified in the discovery set, the miRNA profile could not be confirmed in the subsequent two independent test sets. Our data indicate that there is likely no single, strongly predictive profile of outcome, and even small imbalances with regard to prognostic factors between the recurrent/non-recurrent groups, such as number of tumor-infiltrated lymph nodes at time of diagnosis, may mask identification of potentially predictive miRNAs. Further, it may be speculated that endocrine resistance occurs as a result of several different mechanism, each employing a set of unique miRNA, complicating the identification of a common set of miRNAs that is altered in primary tumors of breast cancer patients who do not have the benefit of adjuvant Tamoxifen treatment.

This finding is somewhat surprising, since several miRNAs have been suggested to be associated with Tamoxifen resistance. However, the majorities of these studies were performed in cell line models or used very limited numbers of patient samples. Moreover, several studies identified miRNAs involved in ER regulation, and thus only indirectly proposed to be involved in Tamoxifen resistance. One of these, the miR-221/-222 cluster, has been found to be in a feedback loop with ERα [21] that results in a decrease in ERα protein, which can lead to resistance towards Tamoxifen. The reason for not identifying this miRNA cluster in our study may be related to the fact that most of the patient tumor samples we analyzed exhibited a very high percentage of ERα positive cells, with a median of 83% (IHC), across the three sets (Table 1). Another miRNA, miR-210, was identified in a dataset of untreated patients consisting of 25 ER-, 40 ER+ and 8 ER-unknown tumor samples, and therefore likely represents a general marker for outcome [16], whereas our study focused on resistance towards Tamoxifen from the onset. Another recent large study (>100 patient samples) could also not confirm the association of miR-210 with outcome [22].

The discovery set was clinically very homogenous, and the N- and R-groups were nearly identical according to the strong prognostic factors that influence the likelihood of developing recurrence. The two test sets, on the other hand, had more variable clinical characteristics per group. The most significant characteristic in test set #1 was the difference in tumor-infiltrated lymph nodes at time of diagnosis, where the R-group had an average of 8.6, whereas the N-group had 3.0. For test set #2, there was a minor difference in the average tumor size and in tumor-infiltrated lymph nodes, but there was also variation in the duration of treatment with Tamoxifen. The two test sets were randomly selected, as opposed to the discovery set, which was matched, and potential biomarkers, such as miRNAs, would need the capacity to overcome patient-to-patient variation (random characteristics) to achieve clinical value. Thus, the miRNA profile does not seem to provide information with regard to the probability of recurrence following adjuvant Tamoxifen-treatment in post-menopausal ER+ breast cancer patients.

The only significant association with a clinical parameter in the discovery set was miR-7, which was associated with tumor grade (p = 0.02, Figure 3). This miRNA has been found to target, amongst others, the *EGFR* [23], [24] and *IGF1R* [25]. Another study on lung cancer found miR-7 to be an oncogenic miRNA, as EGFR was found to induce miR-7 expression through a Ras/ERK/Myc pathway, which promoted cell growth and tumor formation [26], indicating an indirect feedback loop. The expression of the EGFR has also been negatively associated with the expression of miR-30c, which was, identified as an independent predictor of outcome in advanced breast cancer; high expression of miR-30c was associated with benefit of Tamoxifen [27]. Cross-talk between ER and EGFR and its family of growth factor receptors, especially in its activated phosphorylated form, has also been shown to be strongly associated with Tamoxifen resistance [28], [29]. We previously conducted a study of mRNA expression in patient samples from the discovery set and found no significant association between *EGFR* mRNA levels and tumor grade, but a significant association with outcome (p = 0.022) [30]. Furthermore, using the same patient material (discovery set), a significant correlation between the level of pEGFR and outcome was also observed on a tissue microarray (TMA) [28], but again, no correlation with grade was observed. It is well-known that tumor grade is a predictor of poor outcome [31]. The cause of this lack of consistency between the miRNA and mRNA studies with regards to associations to outcome may reflect the complexity of the biological system with multiple miRNAs per target, and that miRNAs have several targets. Furthermore, the apparent indirect feedback loop between miR-7 and EGFR is affected by several other factors, likely masking the known association with grade to outcome.

To investigate whether there were underlying biological associations within the tumor samples, we conducted an unsupervised cluster analysis for each of the three patient sample sets. The miRNAs that exhibited the most variable expression in the individual set and overlapped across the three sets were selected, yielded a set of 31 miRNAs (Table S1). Since the discussed miRNAs were identified by unsupervised analysis, whether they merely depict the cellular architecture of the tumors or are actually masked markers for the tumors ability to progress despite adjuvant treatment with a target-specific drug remains to be determined. One fourth of the 31 miRNAs were found to be associated with EMT/MET and stem cell characteristics, and the notion that cancer stem cells play an important role in endocrine resistance has recently been discussed [32]. Further studies into this field are needed. It would also be interesting to conduct *in situ* hybridization of the identified miRNAs to visualize the cells they are expressed in. Also, functional studies would clarify whether targeting surrounding tissue would induce tumor regression, since it is well-known that the microenvironment and malignant cells interact.

Overall, the number of patients in this study provides a strong foundation for the debate on miRNAs and benefit of Tamoxifen in post-menopausal ER+ breast cancer patients. This global analysis, employing the microarray-technique, allows a general estimation of miRNAs potential and is a common hypothesis-generating method. This also implies, however, that the significance of single miRNAs expressed by a subgroup of patients with yet unknown characteristics may be masked when assessing the groups collectedly as only general differences are detected.

In conclusion, our data seems to indicate that no single miRNA profile predictive of outcome for tumors from ER+ breast cancer patients receiving adjuvant Tamoxifen mono-therapy can be identified. Our study also highlights the difficulty in identifying prognostic/predictive markers in a population of patients with relatively good prognoses. Identification of the few “poor prognostic cells” may be difficult, as their signal may be overshadowed by the majority of cells with “good prognosis”, potentially masking the sought-after biomarkers.

## Supporting Information

Figure S1 **Heat-map of the most significantly differentially expressed miRNAs associated with outcome after adjuvant Tamoxifen treatment in the A) Test set#1: p<0.01, FDR 24%, variance >0.1, and B) Test set#2: p<0.01, FDR 35% and variance >0.1.** The green symbols above the heat-map indicate samples from patients with no recurrence, whereas the red symbols indicate samples from patients with recurrence. The heat-maps are standardized intensity plots with the intensities ranging from −2 (green) to +2 (red).
(PDF)

Figure S2 **Kaplan-Meier plots of the 10 miRNAs identified in the discovery set distinguishing good- and poor-prognostic patient groups, applied for Test set#1.** A) Probability of recurrence. B) Probability of overall survival.
(PDF)

Figure S3 **Kaplan-Meier plots of the 10 miRNAs identified in the discovery set distinguishing good- and poor-prognostic patient groups, applied for Test set#2.** A) Probability of recurrence. B) Probability of overall survival.
(PDF)

Table S1 miRNAs identified across the three datasets of a total of 152 patient samples, based on the top 100 most variable miRNAs per set. The table is focused on general characteristics and/or the relation to breast cancer. Source: www.mir2disease.org [19].
(PDF)

## References

[1] Blamey RW (2002). Guidelines on endocrine therapy of breast cancer EUSOMA.. Eur J Cancer.

[2] Davies C, Godwin J, Gray R, Clarke M, Cutter D (2011). Relevance of breast cancer hormone receptors and other factors to the efficacy of adjuvant tamoxifen: patient-level meta-analysis of randomised trials.. Lancet.

[3] Baum M, Budzar AU, Cuzick J, Forbes J, Houghton JH (2002). Anastrozole alone or in combination with tamoxifen versus tamoxifen alone for adjuvant treatment of postmenopausal women with early breast cancer: first results of the ATAC randomised trial.. Lancet.

[4] Coates AS, Keshaviah A, Thurlimann B, Mouridsen H, Mauriac L (2007). Five years of letrozole compared with tamoxifen as initial adjuvant therapy for postmenopausal women with endocrine-responsive early breast cancer: update of study BIG 1–98.. J Clin Oncol.

[5] Mouridsen HT, Giobbie-Hurder A, Mauriac L, Paridaens R, Colleoni M (2008). BIG 1–98: A randomized double-blind phase III study evaluating letrozole and tamoxifen given in sequence as adjuvant endocrine therapy for postmenopausal women with receptor-positive breast cancer. SABCS, abstract 13: abstracts2view.com/sabcs/view.php?. nu = SABCS08L_.

[6] Esquela-Kerscher A, Slack FJ (2006). Oncomirs - microRNAs with a role in cancer.. Nat Rev Cancer.

[7] Calin GA, Sevignani C, Dumitru CD, Hyslop T, Noch E (2004). Human microRNA genes are frequently located at fragile sites and genomic regions involved in cancers.. Proc Natl Acad Sci U S A.

[8] Adams BD, Furneaux H, White BA (2007). The micro-ribonucleic acid (miRNA) miR-206 targets the human estrogen receptor-alpha (ERalpha) and represses ERalpha messenger RNA and protein expression in breast cancer cell lines.. Mol Endocrinol.

[9] Tessel MA, Krett NL, Rosen ST (2010). Steroid receptor and microRNA regulation in cancer..

[10] Pogribny IP, Tryndyak VP, Boyko A, Rodriguez-Juarez R, Beland FA (2007). Induction of microRNAome deregulation in rat liver by long-term tamoxifen exposure.. Mutat Res.

[11] Masri S, Liu Z, Phung S, Wang E, Yuan YC (2010). The role of microRNA-128a in regulating TGFbeta signaling in letrozole-resistant breast cancer cells.. Breast Cancer Res Treat.

[12] Miller TE, Ghoshal K, Ramaswamy B, Roy S, Datta J (2008). MicroRNA-221/222 confers tamoxifen resistance in breast cancer by targeting p27Kip1.. J Biol Chem.

[13] Cittelly DM, Das PM, Salvo VA, Fonseca JP, Burow ME (2010). Oncogenic HER2{Delta}16 suppresses miR-15a/16 and deregulates BCL-2 to promote endocrine resistance of breast tumors.. Carcinogenesis.

[14] Sachdeva M, Wu H, Ru P, Hwang L, Trieu V (2011). MicroRNA-101-mediated Akt activation and estrogen-independent growth.. Oncogene.

[15] McShane LM, Altman DG, Sauerbrei W, Taube SE, Gion M (2005). Reporting recommendations for tumor marker prognostic studies (REMARK).. J Natl Cancer Inst.

[16] Rothe F, Ignatiadis M, Chaboteaux C, Haibe-Kains B, Kheddoumi N (2011). Global MicroRNA Expression Profiling Identifies MiR-210 Associated with Tumor Proliferation, Invasion and Poor Clinical Outcome in Breast Cancer.. PLoS One.

[17] Maillot G, Lacroix-Triki M, Pierredon S, Gratadou L, Schmidt S (2009). Widespread estrogen-dependent repression of micrornas involved in breast tumor cell growth.. Cancer Res.

[18] Moller S, Jensen MB, Ejlertsen B, Bjerre KD, Larsen M (2008). The clinical database and the treatment guidelines of the Danish Breast Cancer Cooperative Group (DBCG); its 30-years experience and future promise.. Acta Oncol.

[19] Jiang Q, Wang Y, Hao Y, Juan L, Teng M (2009). miR2Disease: a manually curated database for microRNA deregulation in human disease.. Nucleic Acids Res.

[20] Andersen J, Kamby C, Ejlertsen B, Cold S, Ewertz M (2008). Tamoxifen for one year versus two years versus 6 months of Tamoxifen and 6 months of megestrol acetate: a randomized comparison in postmenopausal patients with high-risk breast cancer (DBCG 89C).. Acta Oncol.

[21] Zhao JJ, Lin J, Yang H, Kong W, He L (2008). MicroRNA-221/222 negatively regulates estrogen receptor alpha and is associated with tamoxifen resistance in breast cancer.. J Biol Chem.

[22] Farazi TA, Horlings HM, Ten Hoeve JJ, Mihailovic A, Halfwerk H (2011). MicroRNA sequence and expression analysis in breast tumors by deep sequencing.. Cancer Res.

[23] Webster RJ, Giles KM, Price KJ, Zhang PM, Mattick JS (2009). Regulation of epidermal growth factor receptor signaling in human cancer cells by microRNA-7.. J Biol Chem.

[24] Kefas B, Godlewski J, Comeau L, Li Y, Abounader R (2008). microRNA-7 inhibits the epidermal growth factor receptor and the Akt pathway and is down-regulated in glioblastoma.. Cancer Res.

[25] Jiang L, Liu X, Chen Z, Jin Y, Heidbreder CE (2010). MicroRNA-7 targets IGF1R (insulin-like growth factor 1 receptor) in tongue squamous cell carcinoma cells.. Biochem J.

[26] Chou YT, Lin HH, Lien YC, Wang YH, Hong CF (2010). EGFR promotes lung tumorigenesis by activating miR-7 through a Ras/ERK/Myc pathway that targets the Ets2 transcriptional repressor ERF.. Cancer Res.

[27] Rodriguez-Gonzalez FG, Sieuwerts AM, Smid M, Look MP, Meijer-van Gelder ME (2010). MicroRNA-30c expression level is an independent predictor of clinical benefit of endocrine therapy in advanced estrogen receptor positive breast cancer..

[28] Frogne T, Laenkholm AV, Lyng MB, Henriksen KL, Lykkesfeldt AE (2009). Determination of HER2 phosphorylation at tyrosine 1221/1222 improves prediction of poor survival for breast cancer patients with hormone receptor-positive tumors.. Breast Cancer Res.

[29] Citri A, Yarden Y (2006). EGF-ERBB signalling: towards the systems level.. Nat Rev Mol Cell Biol.

[30] Lyng MB, Lænkholm AV, Tan Q, Vach W, Gravgaard KH (2011). Prediction of outcome of Tamoxifen-treated estrogen receptor-positive, high-risk, primary breast cancer patients as determined by focused gene expression signature (*Submitted*).

[31] Elston CW, Ellis IO (2002). Pathological prognostic factors in breast cancer..

[32] O’Brien CS, Howell SJ, Farnie G, Clarke RB (2009). Resistance to endocrine therapy: are breast cancer stem cells the culprits?. J Mammary Gland Biol Neoplasia.

