# Supplementary material for: Global MicroRNA Expression Profiling of High-Risk ER+ Breast Cancers from Patients Receiving Adjuvant Tamoxifen Mono-Therapy: A DBCG Study
Source: PLoS One. 2012 May 18;7(5):e36170. doi: 10.1371/journal.pone.0036170 (PMC3356496; doi:10.1371/journal.pone.0036170)

Figure S1

Heat-map of the most significantly differentially expressed miRNAs associated with outcome after adjuvant Tamoxifen treatment in the A) Test set#1: p<0.01, FDR 24 %, variance >0.1, and B) Test set#2: p<0.01, FDR 35 % and variance >0.1. The green symbols above the heat-map indicate samples from patients with no recurrence, whereas the red symbols indicate samples from patients with recurrence. The heat-maps are standardized intensity plots with the intensities ranging from -2 (green) to +2 (red).

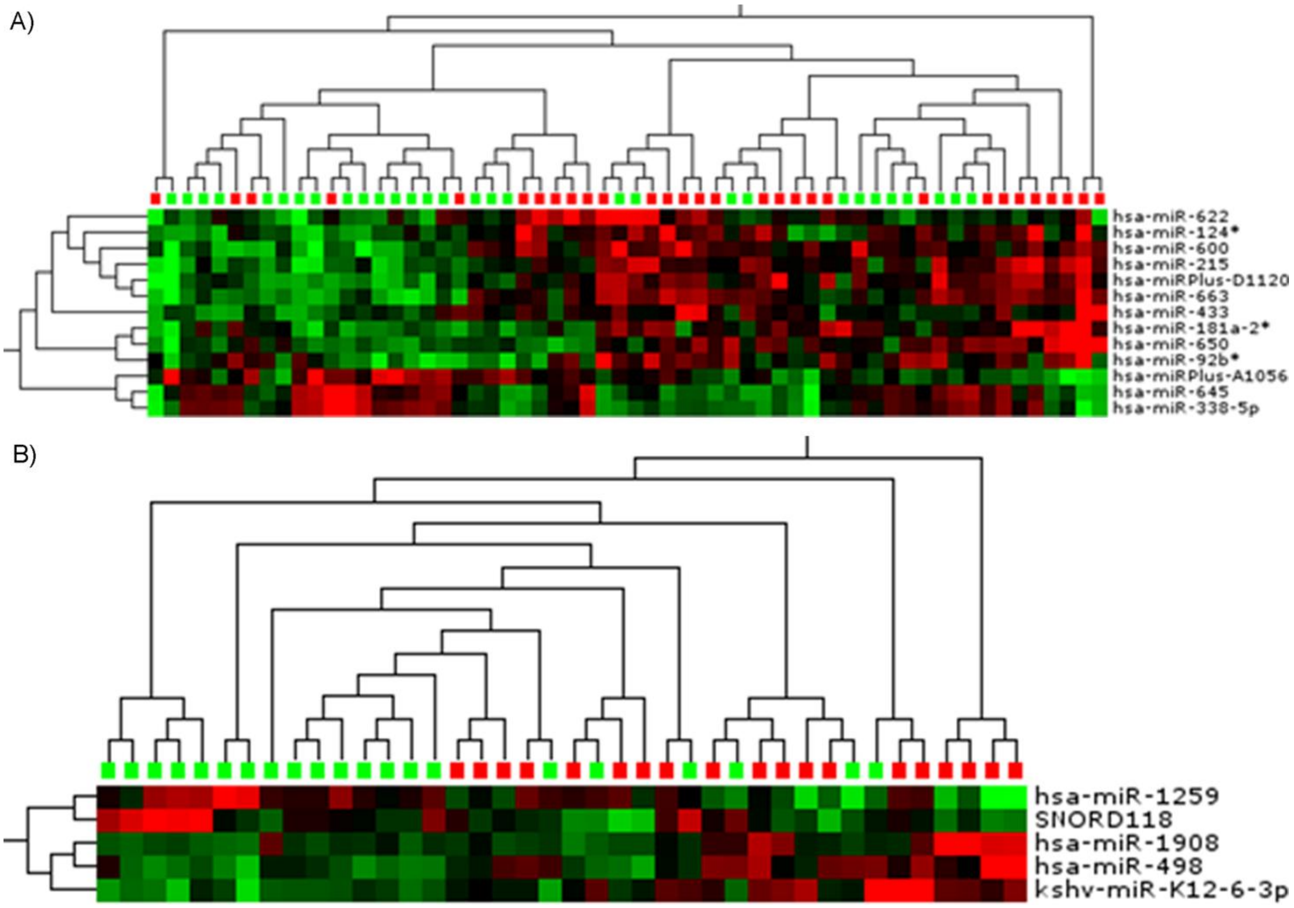

Supplement: Figure S1 — Heat-map of the most significantly differentially expressed miRNAs associated with outcome after adjuvant Tamoxifen treatment in the A) Test set#1: p<0.01, FDR 24%, variance >0.1, and B) Test set#2: p<0.01, FDR 35% and variance >0.1. The green symbols above the heat-map indicate samples from patients with no recurrence, whereas the red symbols indicate samples from patients with recurrence. The heat-maps are standardized intensity plots with the intensities ranging from −2 (green) to +2 (red). (PDF) [file pone.0036170.s001.pdf]
