# Supplementary material for: Global MicroRNA Expression Profiling of High-Risk ER+ Breast Cancers from Patients Receiving Adjuvant Tamoxifen Mono-Therapy: A DBCG Study
Source: PLoS One. 2012 May 18;7(5):e36170. doi: 10.1371/journal.pone.0036170 (PMC3356496; doi:10.1371/journal.pone.0036170)

Figure S2

Kaplan-Meier plots of the 10 miRNAs identified in the discovery set distinguishing good- and poor-prognostic patient groups, applied for Test set#1.

A) Probability of recurrence. B) Probability of overall survival.

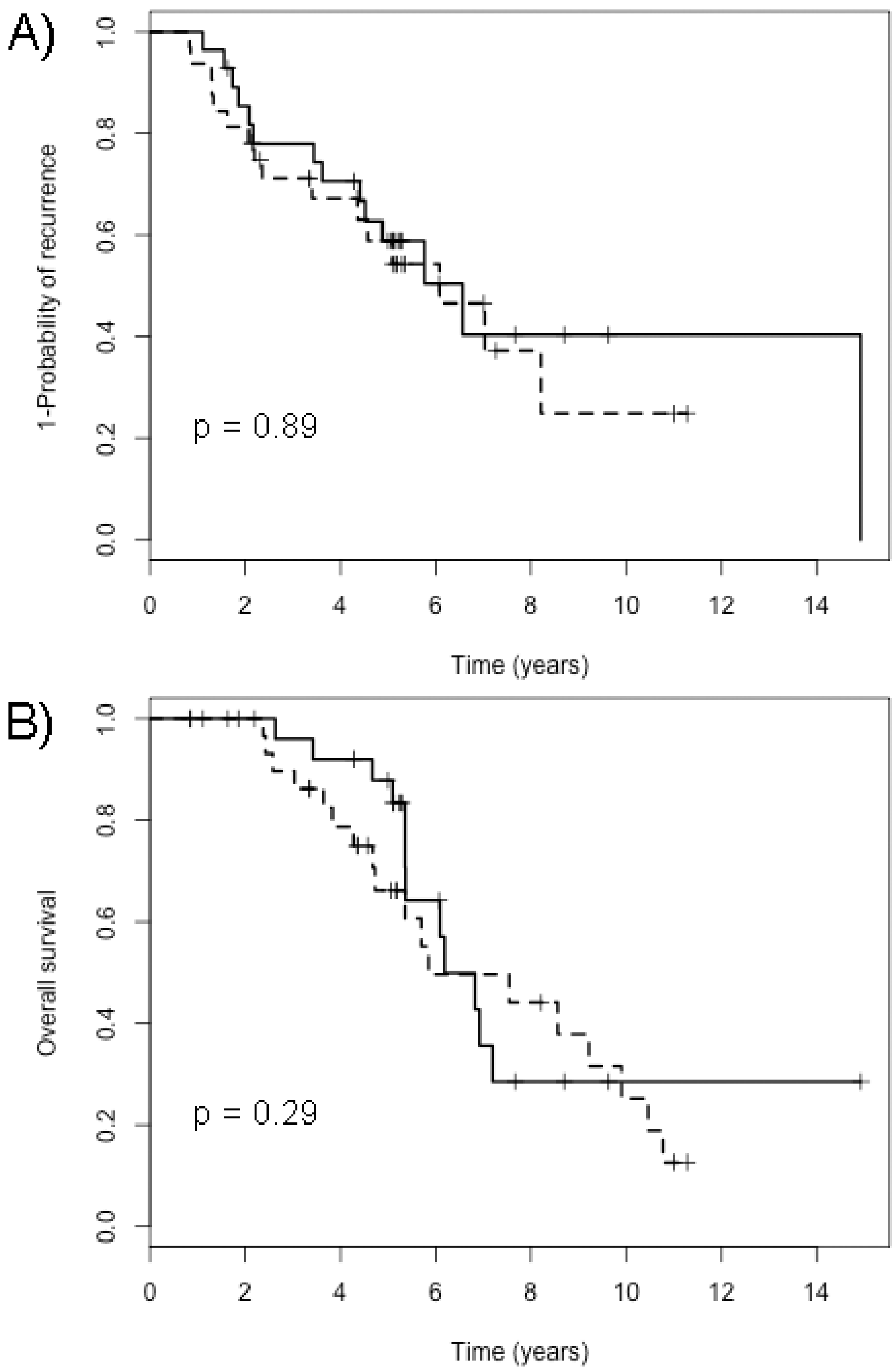

Supplement: Figure S2 — Kaplan-Meier plots of the 10 miRNAs identified in the discovery set distinguishing good- and poor-prognostic patient groups, applied for Test set#1. A) Probability of recurrence. B) Probability of overall survival. (PDF) [file pone.0036170.s002.pdf]
