# Supplementary material for: Global MicroRNA Expression Profiling of High-Risk ER+ Breast Cancers from Patients Receiving Adjuvant Tamoxifen Mono-Therapy: A DBCG Study
Source: PLoS One. 2012 May 18;7(5):e36170. doi: 10.1371/journal.pone.0036170 (PMC3356496; doi:10.1371/journal.pone.0036170)
Supplement: Table S1 — miRNAs identified across the three datasets of a total of 152 patient samples, based on the top 100 most variable miRNAs per set. The table is focused on general characteristics and/or the relation to breast cancer. Source: www.mir2disease.org [19]. (PDF) [file pone.0036170.s004.pdf]

Table S1

miRNAs identified across the three datasets of a total of 152 patient samples, based on the top 100 most variable miRNAs per set. The table is focused on general characteristics and/or the relation to breast cancer. Source: www.mir2disease.org [1].

miRNA	Notes	Reference
hsa-let-7a	Self-renewal/tumorgenicity	[2;3]
hsa-let-7b	EMT	[3]
hsa-let-7c	EMT	[2;3]
hsa-miR-10a	Hoxd4,-a1	[4-6]
hsa-miR-125b	Tumor supp./reduces ERBB2 and ERBB3 expr.	[7;8]
hsa-miR-126	IRS-1 (endothelial cells)	[9;10]
hsa-miR-141	EMT/ZEB1, ZEB2	[11;12]
hsa-miR-142-3p	Hematopoietic -specific	[13]
hsa-miR-144	Erythropoiesis	[14]
hsa-miR-193a-3p	Tumor supp. (oral cancer)	[15]
hsa-miR-196a	HOXb8 genes	[16]
hsa-miR-199a-3p/-199b-3p	Microcirculation	[17]
hsa-miR-199a-5p	Microcirculation	[17]
hsa-miR-200a	EMT	[3;11;12]
hsa-miR-200b	EMT	[3;11;12]
hsa-miR-200c	EMT	[3;11;12]
hsa-miR-205	Basal cells/EMT	[3;11;12]
hsa-miR-21	Primarily oncogenic	[18-21]
hsa-miR-22	ER	[22]
hsa-miR-23a	oncogenic	[23]
hsa-miR-29a	Methylation/DNMT3A,-B	[24]
hsa-miR-29b	Methylation/DNMT3A,-B	[24]
hsa-miR-29c	Methylation/DNMT3A,-B, ex.cell matrix proteins/invasiveness	[24;25]
hsa-miR-30a	Autophagy by targeting Beclin 1	[26]
hsa-miR-30b	Regulated by ER	[27]

hsa-miR-30c	Tam resistance	[28]
hsa-miR-342-3p	Tam resistance	[29]
hsa-miR-449a	Cell growth,viability/HDAC1	[30]
hsa-miR-451	Doxorubicin resistance/MDR1, erythrocytes	[31;32]
hsa-miR-886-3p	VTRNA2/multidrug-resistance	[33]
hsa-miR-886-5p	VTRNA2/multidrug-resistance	[33]

References

1. Jiang Q, Wang Y, Hao Y, Juan L, Teng M, Zhang X, Li M, Wang G, Liu Y: **miR2Disease: a manually curated database for microRNA deregulation in human disease**. *Nucleic Acids Res* 2009, **37:**D98-104.

2. Yu F, Yao H, Zhu P, Zhang X, Pan Q, Gong C, Huang Y, Hu X, Su F, Lieberman J, Song E: **let-7 regulates self renewal and tumorigenicity of breast cancer cells**. *Cell* 2007, **131:**1109-1123.

3. Peter ME: **Let-7 and miR-200 microRNAs: guardians against pluripotency and cancer progression**. *Cell Cycle* 2009, **8:**843-852.

4. Tan Y, Zhang B, Wu T, Skogerbo G, Zhu X, Guo X, He S, Chen R: **Transcriptional inhibiton of Hoxd4 expression by miRNA-10a in human breast cancer cells**. *BMC.Mol.Biol.* 2009, **10:**12.

5. Garzon R, Pichiorri F, Palumbo T, Iuliano R, Cimmino A, Aqeilan R, Volinia S, Bhatt D, Alder H, Marcucci G, Calin GA, Liu CG, Bloomfield CD, Andreeff M, Croce CM: **MicroRNA fingerprints during human megakaryocytopoiesis**. *Proc.Natl.Acad.Sci.U.S.A* 2006, **103:**5078-5083.

6. Weiss FU, Marques IJ, Woltering JM, Vlecken DH, Aghdassi A, Partecke LI, Heidecke CD, Lerch MM, Bagowski CP: **Retinoic acid receptor antagonists inhibit miR-10a expression and block metastatic behavior of pancreatic cancer**. *Gastroenterology* 2009, **137:**2136-2145.

7. Iorio MV, Ferracin M, Liu CG, Veronese A, Spizzo R, Sabbioni S, Magri E, Pedriali M, Fabbri M, Campiglio M, Menard S, Palazzo JP, Rosenberg A, Musiani P, Volinia S, Nenci I, Calin GA, Querzoli P, Negrini M, Croce CM: **MicroRNA gene expression deregulation in human breast cancer**. *Cancer Res* 2005, **65:**7065-7070.

8. Scott GK, Goga A, Bhaumik D, Berger CE, Sullivan CS, Benz CC: **Coordinate suppression of ERBB2 and ERBB3 by enforced expression of micro-RNA miR-125a or miR-125b**. *J.Biol.Chem.* 2007, **282:**1479-1486.

9. Tavazoie SF, Alarcon C, Oskarsson T, Padua D, Wang Q, Bos PD, Gerald WL, Massague J: **Endogenous human microRNAs that suppress breast cancer metastasis**. *Nature* 2008, **451:**147-152.

10. Zhang J, Du YY, Lin YF, Chen YT, Yang L, Wang HJ, Ma D: **The cell growth suppressor, mir-126, targets IRS-1**. *Biochem.Biophys.Res Commun.* 2008, **377:**136-140.

11. Gregory PA, Bert AG, Paterson EL, Barry SC, Tsykin A, Farshid G, Vadas MA, Khew-Goodall Y, Goodall GJ: **The miR-200 family and miR-205 regulate epithelial to mesenchymal transition by targeting ZEB1 and SIP1**. *Nat.Cell Biol.* 2008, **10:**593-601.

12. Burk U, Schubert J, Wellner U, Schmalhofer O, Vincan E, Spaderna S, Brabletz T: **A reciprocal repression between ZEB1 and members of the miR-200 family promotes EMT and invasion in cancer cells**. *EMBO Rep.* 2008, **9:**582-589.

13. Ramkissoon SH, Mainwaring LA, Ogasawara Y, Keyvanfar K, McCoy JP, Jr., Sloand EM, Kajigaya S, Young NS: **Hematopoietic-specific microRNA expression in human cells**. *Leuk.Res* 2006, **30:**643-647.

14. Fu YF, Du TT, Dong M, Zhu KY, Jing CB, Zhang Y, Wang L, Fan HB, Chen Y, Jin Y, Yue GP, Chen SJ, Chen Z, Huang QH, Jing Q, Deng M, Liu TX: **Mir-144 selectively regulates embryonic alpha-hemoglobin synthesis during primitive erythropoiesis**. *Blood* 2009, **113:**1340-1349.

15. Kozaki K, Imoto I, Mogi S, Omura K, Inazawa J: **Exploration of tumor-suppressive microRNAs silenced by DNA hypermethylation in oral cancer**. *Cancer Res* 2008, **68:**2094-2105.

16. Mansfield JH, Harfe BD, Nissen R, Obenauer J, Srineel J, Chaudhuri A, Farzan-Kashani R, Zuker M, Pasquinelli AE, Ruvkun G, Sharp PA, Tabin CJ, McManus MT: **MicroRNA-responsive 'sensor' transgenes uncover Hox-like and other developmentally regulated patterns of vertebrate microRNA expression**. *Nat.Genet.* 2004, **36:**1079-1083.

17. Yeligar S, Tsukamoto H, Kalra VK: **Ethanol-induced expression of ET-1 and ET-BR in liver sinusoidal endothelial cells and human endothelial cells involves hypoxia-inducible factor-1alpha and microrNA-199**. *J.Immunol.* 2009, **183:**5232-5243.

18. Yan LX, Huang XF, Shao Q, Huang MY, Deng L, Wu QL, Zeng YX, Shao JY: **MicroRNA miR-21 overexpression in human breast cancer is associated with advanced clinical stage, lymph node metastasis and patient poor prognosis**. *RNA.* 2008, **14:**2348-2360.

19. Huang TH, Wu F, Loeb GB, Hsu R, Heidersbach A, Brincat A, Horiuchi D, Lebbink RJ, Mo YY, Goga A, McManus MT: **Up-regulation of miR-21 by HER2/neu signaling promotes cell invasion**. *J.Biol.Chem.* 2009, **284:**18515-18524.

20. Zhu S, Wu H, Wu F, Nie D, Sheng S, Mo YY: **MicroRNA-21 targets tumor suppressor genes in invasion and metastasis**. *Cell Res* 2008, **18:**350-359.

21. Miller TE, Ghoshal K, Ramaswamy B, Roy S, Datta J, Shapiro CL, Jacob S, Majumder S: **MicroRNA-221/222 confers tamoxifen resistance in breast cancer by targeting p27Kip1**. *J Biol.Chem.* 2008, **283:**29897-29903.

22. Pandey DP, Picard D: **miR-22 inhibits estrogen signaling by directly targeting the estrogen receptor alpha mRNA**. *Mol.Cell Biol.* 2009, **29:**3783-3790.

23. Huang S, He X, Ding J, Liang L, Zhao Y, Zhang Z, Yao X, Pan Z, Zhang P, Li J, Wan D, Gu J: **Upregulation of miR-23a approximately 27a approximately 24 decreases transforming growth factor-beta-induced tumor-suppressive activities in human hepatocellular carcinoma cells**. *Int.J.Cancer* 2008, **123:**972-978.

24. Fabbri M, Garzon R, Cimmino A, Liu Z, Zanesi N, Callegari E, Liu S, Alder H, Costinean S, Fernandez-Cymering C, Volinia S, Guler G, Morrison CD, Chan KK, Marcucci G, Calin GA, Huebner K, Croce CM: **MicroRNA-29 family reverts aberrant methylation in lung cancer by targeting DNA methyltransferases 3A and 3B**. *Proc.Natl.Acad.Sci.U.S.A* 2007, **104:**15805-15810.

25. Sengupta S, den Boon JA, Chen IH, Newton MA, Stanhope SA, Cheng YJ, Chen CJ, Hildesheim A, Sugden B, Ahlquist P: **MicroRNA 29c is down-regulated in nasopharyngeal carcinomas, up-regulating mRNAs encoding extracellular matrix proteins**. *Proc.Natl.Acad.Sci.U.S.A* 2008, **105:**5874-5878.

26. Zhu H, Wu H, Liu X, Li B, Chen Y, Ren X, Liu CG, Yang JM: **Regulation of autophagy by a beclin 1-targeted microRNA, miR-30a, in cancer cells**. *Autophagy.* 2009, **5:**816-823.

27. Mellios N, Galdzicka M, Ginns E, Baker SP, Rogaev E, Xu J, Akbarian S: **Gender-Specific Reduction of Estrogen-Sensitive Small RNA, miR-30b, in Subjects With Schizophrenia**. *Schizophr.Bull.* 2010.

28. Rodriguez-Gonzalez FG, Sieuwerts AM, Smid M, Look MP, Meijer-van Gelder ME, de W, V, Sleijfer S, Martens JW, Foekens JA: **MicroRNA-30c expression level is an independent predictor of clinical benefit of endocrine therapy in advanced estrogen receptor positive breast cancer**. *Breast Cancer Res.Treat.* 2010.

29. Cittelly DM, Das PM, Spoelstra NS, Edgerton SM, Richer JK, Thor AD, Jones FE: **Downregulation of miR-342 is associated with tamoxifen resistant breast tumors**. *Mol.Cancer* 2010, **9:**317.

30. Noonan EJ, Place RF, Pookot D, Basak S, Whitson JM, Hirata H, Giardina C, Dahiya R: **miR-449a targets HDAC-1 and induces growth arrest in prostate cancer**. *Oncogene* 2009, **28:**1714-1724.

31. Kovalchuk O, Filkowski J, Meservy J, Ilnytskyy Y, Tryndyak VP, Chekhun VF, Pogribny IP: **Involvement of microRNA-451 in resistance of the MCF-7 breast cancer cells to chemotherapeutic drug doxorubicin**. *Mol.Cancer Ther.* 2008, **7:**2152-2159.

32. Sempere LF, Christensen M, Silahtaroglu A, Bak M, Heath CV, Schwartz G, Wells W, Kauppinen S, Cole CN: **Altered MicroRNA expression confined to specific epithelial cell subpopulations in breast cancer**. *Cancer Res* 2007, **67:**11612-11620.

33. Nandy C, Mrazek J, Stoiber H, Grasser FA, Huttenhofer A, Polacek N: **Epstein-barr virus-induced expression of a novel human vault RNA**. *J.Mol.Biol.* 2009, **388:**776-784.
